# Newly diagnosed glioblastoma in geriatric (65 +) patients: impact of patients frailty, comorbidity burden and obesity on overall survival

**DOI:** 10.1007/s11060-020-03625-2

**Published:** 2020-09-29

**Authors:** Matthias Schneider, Anna-Laura Potthoff, Elisa Scharnböck, Muriel Heimann, Niklas Schäfer, Johannes Weller, Christina Schaub, Andreas H. Jacobs, Erdem Güresir, Ulrich Herrlinger, Hartmut Vatter, Patrick Schuss

**Affiliations:** 1 Department of Neurosurgery, University Hospital Bonn, Venusberg-Campus 1, 53127 Bonn, Germany; 2 Division of Clinical Neurooncology, Department of Neurology, University Hospital Bonn, Bonn, Germany; 3 Department of Geriatric Medicine and Neurology, Johanniterkrankenhaus and CIO Bonn, Bonn, Germany

**Keywords:** Geriatric glioblastoma patients, Frailty, Comorbidity, Survival

## Abstract

**Object:**

Increasing age is a known negative prognostic factor for glioblastoma. However, a multifactorial approach is necessary to achieve optimal neuro-oncological treatment. It remains unclear to what extent frailty, comorbidity burden, and obesity might exert influence on survival in geriatric glioblastoma patients. We have therefore reviewed our institutional database to assess the prognostic value of these factors in elderly glioblastoma patients.

**Methods:**

Between 2012 and 2018, patients aged ≥ 65 years with newly diagnosed glioblastoma were included in this retrospective analysis. Patients frailty was analyzed using the modified frailty index (mFI), while patients comorbidity burden was assessed according to the Charlson comorbidity index (CCI). Body mass index (BMI) was used as categorized variable.

**Results:**

A total of 110 geriatric patients with newly diagnosed glioblastoma were identified. Geriatric patients categorized as least-frail achieved a median overall survival (mOS) of 17 months, whereas most frail patients achieved a mOS of 8 months (p = 0.003). Patients with a CCI > 2 had a lower mOS of 6 months compared to patients with a lower comorbidity burden (12 months; p = 0.03). Multivariate analysis identified “subtotal resection” (p = 0.02), “unmethylated MGMT promoter status” (p = 0.03), “BMI < 30” (p = 0.04), and “frail patient (mFI ≥ 0.27)” (p = 0.03) as significant and independent predictors of 1-year mortality in geriatric patients with surgical treatment of glioblastoma (Nagelkerke's R^2^ 0.31).

**Conclusions:**

The present study concludes that both increased frailty and comorbidity burden are significantly associated with poor OS in geriatric patients with glioblastoma. Further, the present series suggests an obesity paradox in geriatric glioblastoma patients.

**Electronic supplementary material:**

The online version of this article (10.1007/s11060-020-03625-2) contains supplementary material, which is available to authorized users.

## Introduction

Although maximally safe surgery and adjuvant therapy have been the standard of care in glioblastoma treatment for many years, the management of elderly patients remains a challenge due to the increased incidence of treatment-related toxicities and slower recovery rates [1–5].

The challenge for optimal treatment of glioblastoma in geriatric patients resides in the balance between maximum radicality and reduction of intervention-related adverse events, which must be determined individually. Therefore, for elderly patients, specific aspects are important for a better assessment of treatment progression/success, such as a more detailed assessment of physical resources and function before each treatment.

There is scarce data available on health status, physical resources, frailty and comorbidity burden as well as their implications for survival in older patients with glioblastoma.

Therefore, we have analyzed our institutional database with regard to a potential impact of the abovementioned pre-, peri- as well as immediately postoperatively collectable parameters on the success of treatment in geriatric patients with newly diagnosed glioblastoma.

## Materials and methods

### Patients

All patients with newly diagnosed glioblastoma who were operated on at the authors' facility between 2012 and 2018 were entered into a computerized database (SPSS, version 25, IBM Corp., Armonk, NY). Only patients aged ≥ 65 years who underwent surgical resection for newly diagnosed glioblastoma were included in the further analysis. Approval for this study was granted by the institutional ethics committee.

Information, including patient characteristics, radiological features, methylation status of the MGMT promoter, body mass index (BMI), functional neurological status at admission and during the course of treatment was recorded and further analyzed. The Karnofsky performance score (KPS) was used to evaluate geriatric patients according to their neurological functional status. In this context, KPS ≥ 70 was defined as a favorable outcome during postoperative follow-up immediately after surgery and 3 and 12 months postoperatively. Patients frailty was analyzed using the modified frailty index (mFI), while patients comorbidity burden was assessed according to the Charlson comorbidity index (CCI).

Treatment decisions were made at the initial presentation of the patient and during follow-up by the institutional interdisciplinary tumor board meetings of the Center of Neurooncology, as described previously [6].

The extent of resection (EOR) was assessed in early (< 72 h) postoperative magnetic resonance imaging (MRI, 3 T). Gross-total resection (GTR) was determined as complete removal of the contrast-enhancing tissue (i.e. absence of residual enhancing tumor tissue).

Overall survival (OS) was measured from the day of glioblastoma surgery until death or last observation. All parameters were compared in terms of OS.

### Modified frailty index and CCI

The Canadian Study on Health and Aging has developed a standardized frailty index (CSHA–FI) based on a cumulative deficit model. The CSHA–FI was linked to eleven variables from the database of the American Surgeons National Surgical Quality Improvement Program (NSQIP) to create a modified frailty index (mFI) [7]. The mFI thus contains the following 11 items: diabetes; functional status (not independent); chronic obstructive pulmonary disease or pneumonia; congestive heart failure; history of myocardial infarction; hypertension requiring medication; peripheral vascular disease or resting pain; impaired sensory function; transient ischaemic attack or cerebrovascular event in the medical history; previous cerebrovascular accident with neurological deficit; previous percutaneous coronary intervention, previous coronary surgery, or angina pectoris. For the calculation of the index, each item was allocated the same weight (1 point). The mFI was then computed for a given individual patient with total points as the sum of all items divided by 11 [8]. Although the mFI is not intended to be a dichotomized variable, patients were divided into three groups according to their mFI based on previous data: “least-frail” (mFI 0–0.08), “moderately-frail” (mFI 0.09–0.26), and “frailest” (mFI ≥ 0.27) [9].

Based on retrospective review of medical records, the preoperative comorbidity burden of included geriatric patients with glioblastoma was indexed by the CCI, as described elsewhere [10]. Data for the mFI and CCI was obtained from medical chart reviews as first listed diagnoses or derived from administrative systems using the 10th revision of the International Statistical Classification of Diseases and Related Health Problems (ICD-10) codes [11]. These codes were manually examined by two coding experts and a general physician for face validity. According to the current literature and for better clinical applicability, the analyzed geriatric patients with glioblastoma were separated into two groups according to their calculated CCI: CCI 0–2 and CCI > 2 [12, 13].

### Statistics

Data analysis was performed using the computer software package SPSS (version 25, IBM Corp., Armonk, NY). Unpaired categorical and binary variables were analyzed in contingency tables using the Fisher’s exact test. The Mann–Whitney U-test was chosen to compare continuous variables as the data were mostly not normally distributed. OS was analyzed by the Kaplan–Meier method using Gehan–Breslow–Wilcoxon test. Results with p < 0.05 were considered statistically significant.

Furthermore, a backward stepwise method was used to construct a multivariate logistic regression model in order to find independent predictors of 1-year mortality in elderly patients with glioblastoma who underwent surgical resection.

## Results

### Patient characteristics

Between 2012 and 2018, a total of 110 geriatric patients underwent surgery for newly diagnosed glioblastoma at the Department of Neurosurgery, University Hospital Bonn. The median age was 72 years (range 65–86 years). Geriatric glioblastoma patients exhibited a median preoperative KPS of 80 (range 50–100). Tumors most frequently involved the temporal lobe (36%), followed by the frontal (28%), parietal (23%), and occipital (11%) regions. Multifocal findings were present in 6 patients (6%). GTR was performed in 66 patients (60%), STR in 44 patients (40%). Median OS for geriatric patients with glioblastoma was 11 months (95% CI 9.4–12.6). Further details are given in Table 1.

**Table 1:** Patient and tumor characteristics

	n = 110
Median age at operation (95% CI)	72 (65–86)
Female sex	46 (42%)
Median preoperative KPS (95% CI)	80 (50–100)
Median BMI (95% CI)	26.1 (19.6–44.1)
CCI 0–2	92 (84%)
CCI > 2	18 (16%)
Median mFI	0.18
Preoperative anticoagulation/antiplatelet medication	51 (46%)
Tumor-related epilepsy	22 (20%)
MGMT methylated	48 (44%)
MGMT unmethylated	57 (52%)
MGMT not available	5 (5%)
IDH wild type	97 (88%)
IDH mutant	7 (6%)
IDH not availbale	6 (6%)
Adjuvant chemotherapy	83 (75%)
Adjuvant radiotherapy	94 (85%)
mOS (months)	11 (95% CI 9.4–12.6)

*BMI* body mass index, *CCI* Charlson comorbidity index, *CI* confidence interval, *IDH* isocitrate dehydrogenase, *KPS* Karnofsky performance score, *mFI* modified frailty index, *MGMT* O-6-methylguanine-DNA methyltransferase, *mOS* median overall survival, *SD* standard deviation

### Influence of body mass index on overall survival

**Fig. 1:**
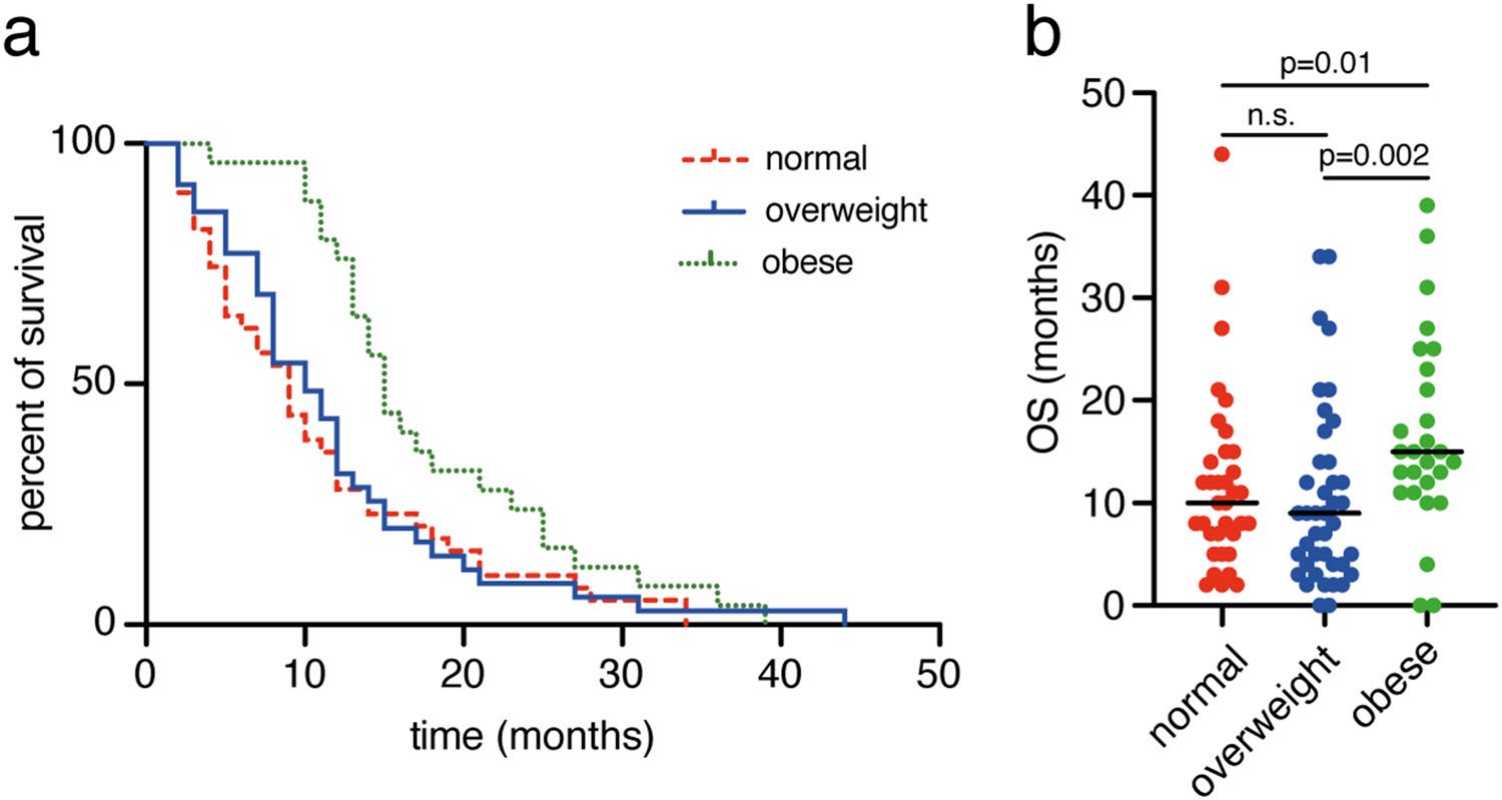
Obesity correlates to prolonged overall survival rates. **a** Kaplan–Meier curves for OS of geriatric glioblastoma patients stratified according to normal weight (BMI 18–24.9 kg/m^2^), overweight (BMI 25–29.9 kg/m^2^) and obese (BMI ≥ 30 kg/m^2^). **b** Scatter plots depict median and distribution of OS dependent on the BMI-levels indicated *BMI* body mass index, *OS* overall survival

Geriatric glioblastoma patients presented with a median BMI of 26.1 (range 19.6–44.1). 39 patients (36%) met the classification of a normal weight (BMI < 25), 43 patients (39%) were overweight (BMI 25–29.9), 28 patients (25%) appeared to be obese (BMI ≥ 30). Patients classified as normal weighted had a mOS of 10 months (95% CI 7.4–12.6), overweight patients had an mOS of 9 months (95% CI 6.4–11.7) and obese patients had a mOS of 15 months (95% CI 12.9–17.1; Fig. 1). Patients with a BMI < 30 had a mPFS of 7 months (95% CI 6–9), patients with a BMI ≥ 30 had a mPFS of 12 (95% CI 7–25; p = 0.036, Supplementary Figure S1a).

### Influence of comorbidity burden on overall survival

**Fig. 2:**
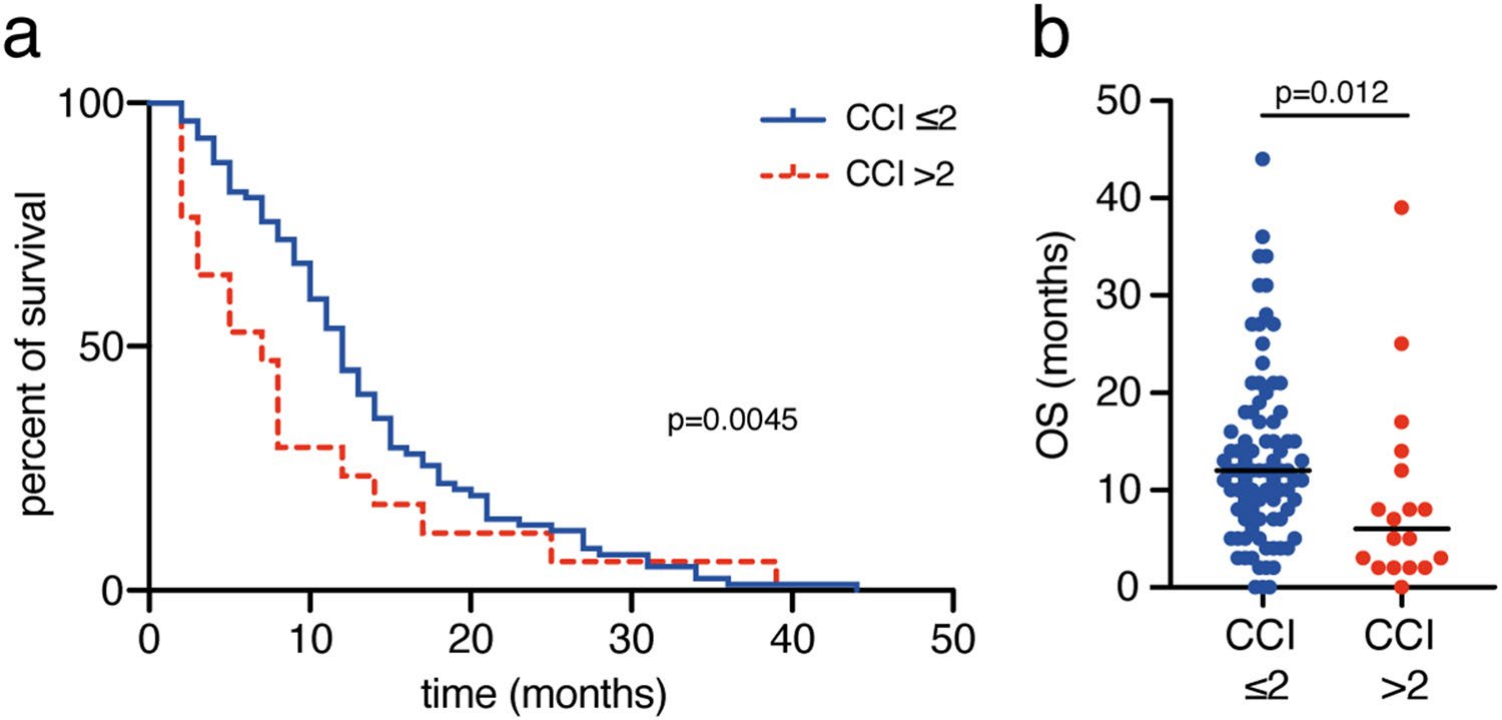
Comorbidity burden correlates to limited overall survival rates. **a** Kaplan–Meier curves for OS stratified into geriatric glioblastoma patients with CCI < 2 and ≤ 2. **b** Scatter plots depict median and distribution of OS dependent on the CCI-levels indicated *CCI* Charlson comorbidity index, *OS* overall survival

Geriatric glioblastoma patients exhibited a median preoperative CCI of 0.5. Detailed frequencies of conditions included in the CCI are given in Table 2. Patients with a CCI ≥ 2 achieved a mOS of 5 months (95% CI 0–10.5), while patients with a lower comorbidity index achieved a mOS of 12 months (95% CI 10.5–13.5; p = 0.012, Fig. 2). Patients with a CCI > 2 achieved a mPFS of 6 months (95% CI 2–12), while patients with a lower comorbidity index achieved a mPFS of 8 months (95% CI 7–12; p = 0.014, Suplementary Figure S1b).

**Table 2:** Frequency of Charlson comorbidity index conditions (n = 110)^a^

Index weight	Condition	Frequency % (n)
1	Coronary artery disease	6.4 (7)
1	Congestive heart failure	3.6 (4)
1	Peripheral vascular disease	0.9 (1)
1	Cerebrovascular disease	7.3 (8)
1	Dementia	5.5 (6)
1	Chronic pulmonary disease	5.5 (6)
1	Connective tissue disease	0 (0)
1	Ulcer disease	2.7 (3)
1	Mild liver disease	1.8 (2)
1	Diabetes	13.6 (15)
2	Hemiplegia	7.3 (8)
2	Moderate/severe renal disease	4.5 (5)
2	Diabetes with end-organ damage	0 (0)
2	Any tumor	16.4 (18)
2	Leukemia	0.9 (1)
2	Lymphoma	0 (0)
3	Moderate/severe liver disease	0.9 (1)
6	Metastatic solid tumor	0.9 (1)
6	AIDS	0 (0)

^a^Values represent number of patients unless otherwise indicated (%)

### Influence of patients frailty on overall survival

**Fig. 3:**
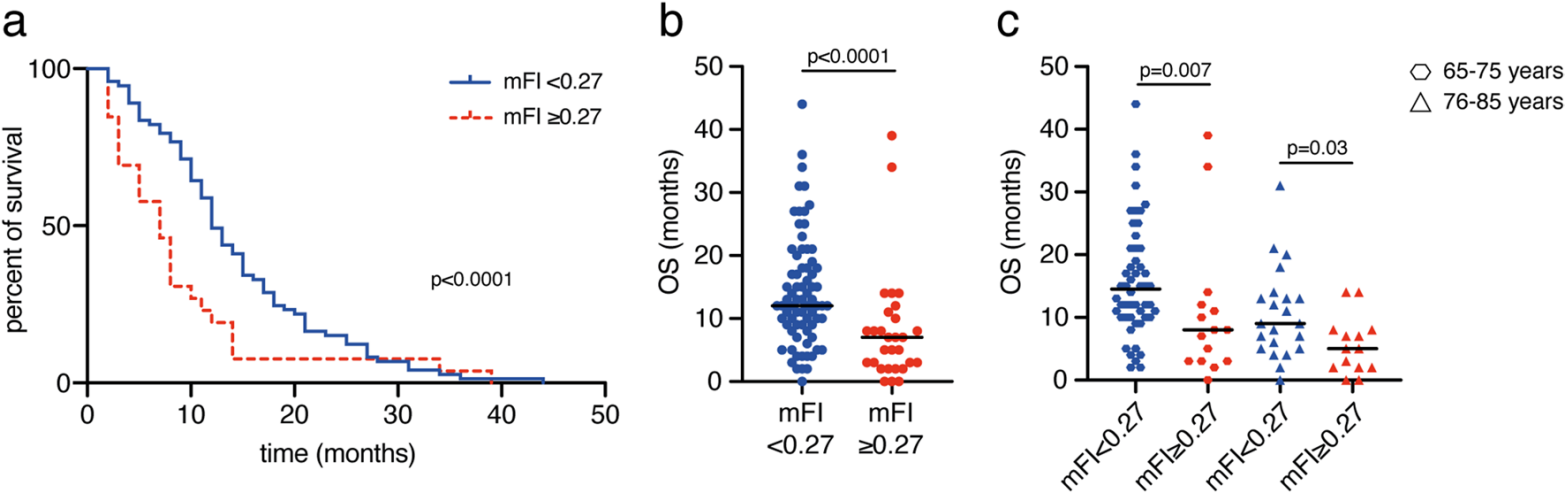
Patient frailty correlates to limited overall survival rates. **a** Kaplan–Meier curves for OS stratified into geriatric glioblastoma patients with mFI < 0.27 and ≥ 0.27. **b** Scatter plots depict median and distribution of OS dependent on the mFI-levels indicated. **c** Scatter plots depict median and distribution of OS dependent on the mFI-levels for further stratification by age as indicated. *mFI* modified frailty index, *OS* overall survival

At preoperative admission status, geriatric glioblastoma patients presented with a median mFI of 0.18. Detailed frequencies of conditions included in the mFI are given in Table 3. Analysis of mortality-rates revealed a considerable higher probability of death with increasing index (Fig. 3). After categorization, geriatric patients defined as least-frail and moderately-frail achieved a mOS of 13 months, whereas the mot frail patients achieved a mOS of 7 months (p < 0.0001, Fig. 3a,b). For the group of patients aged 65–75 years, patients with mFI < 0.27 exhibited a mOS of 16 months, compared to 11 months for patients with mFI ≥ 0.27 (p = 0.007). For the group of patients aged 76–85 years, respective values for mOS were 11 months vs 5.5 months (p = 0.03) (Fig. 3c).

**Table 3:** Frequency of patient frailty according to the modified frailty index (n = 110)

Index weight	Description	Frequency % (n)
1	Functional health status prior surgery (only dependent)	36.4 (40)
1	History of diabetes mellitus	13.6 (15)
1	History of severe COPD/current pneumonia	8.2 (9)
1	Congestive heart failure	3.6 (4)
1	History of myocardial infarction	6.4 (7)
1	Previous percutaneous coronary intervention; previous cardiac surgery; history of angina	34.5 (38)
1	Hypertension requiring medication	67.3 (74)
1	Impaired sensorium	5.5 (6)
1	History of transient ischemic attack	4.5 (5)
1	Cerebrovascular accident/stroke with neurologic deficit	2.7 (3)
1	History of revascularization for peripheral vascular disease	0.9 (1)

Patients with a mFI < 0.27 exhibited a mPFS of 8 months (95% CI 7–12), patients with a mFI ≥ 0.27 had a mPFS of 4 months (95% CI 2–7; p < 0.0004, Supplementary Figure S1c).

### Multivariate analysis

We conducted a multivariate logistic regression analysis to identify independent predictors of 1-year mortality in geriatric patients with glioblastoma. The multivariate analysis identified “subtotal resection” (p = 0.02, OR 3.1, 95% CI 1.2–7.7), “unmethylated MGMT promoter methylation status” (p = 0.03, OR 2.7, 95% CI 1.1–6.5), “BMI < 30” (p = 0.04, OR 3.1, 95% CI 1.1–9.2), and “frail patient classification (mFI ≥ 0.27)” (p = 0.03, OR 3.2, 95% CI 1.1–8.8) as significant and independent predictors of 1-year mortality (Nagelkerke's R^2^ 0.31).

## Discussion

As the incidence of glioblastoma in elderly patients increases as this population grows, it has become increasingly important to identify effective treatment regimens that might extend survival in this vulnerable patient population [14]. Older patients with glioblastoma are often treated more conservatively than younger ones [15]. Several studies have noted that older patients with glioblastoma often only receive biopsy or scaled down adjuvant therapy [15, 16]. Such reduced aggressive treatment management is often ascribed to a potential lack of physical resilience following postoperative complications and/or adjuvant treatment toxicity [17, 18]. In order to sufficiently cope with these advanced challenges in the geriatric patient clientele, it is important to apply and interpret abovementioned established aspects and principles of decision making in the geriatric patient cohort [19, 20]. For this purpose, we have evaluated elderly patients that had undergone comprehensive surgical as well as adjuvant treatment for glioblastoma with regard to the influence of comorbidity burden, frailty and BMI on overall survival.

### Obesity paradox in geriatric patients with glioblastoma

Obesity is a complex condition associated with multiple pathophysiological processes and mechanisms [21]. In the present study, obesity (BMI ≥ 30) was correlated to a survival benefit in geriatric glioblastoma patients. In Contrary to a reported positive influence of elevated BMI levels on survival in patients with glioblastoma averaged over all age groups, various studies provide partially contradictory data [22–24]. This underlines the divergent character of BMI as a measure of obesity in patients with underlying solid malignancies. On the one hand, obesity is associated with a poor prognosis in certain oncological scenarios due to increased circulating concentrations of metabolic and pro-inflammatory hormones that might stimulate tumor growth and metastatic spread [23]. Further hypotheses for the association between obesity and poor outcome include uncontrolled hyperglycemia in obese patients, which may promote tumor growth, and/or suboptimal chemotherapy due to dosage thresholds [22]. On the other hand, it has been suggested that an increased BMI is also associated with a larger muscle mass, which in patients with advanced malignant disease serves as a potent energy source and thus results in a better prognosis [25].

### Influence of patient frailty on survival

In geriatric research, assessing the frailty of elderly patients is an important instrument for predicting morbidity and mortality [26]. In a systematic review of the literature, Pazniokas et al. were able to illustrate that frailty is associated with poor outcomes in a variety of neurosurgical disorders [20]. At the same time, the authors note that the neurosurgical literature is extremely heterogeneous in its methodological assessment of frailty. Following the recommendation of Pazniokas and co-workers, the assessment of frailty in the present study is based on the application of the modified frailty index [7]. Increased frailty of an elderly glioblastoma patient results in a significantly higher probability of poorer survival. We were able to determine the influence of frailty by using the Kaplan–Meier method in terms of overall survival (p = 0.005) as well as applying a multivariate analysis of 1-year mortality (p = 0.03) in our patient cohort.

The relevance of these results, especially with regard to the influence of the frailty of elderly patients with glioblastoma on outcome, should not be underestimated. According to a recent study of Rahmani et al. surgery for glioblastoma in the eldely consitutes a safe treatment modality resulting in low death and life-threatening morbidity [27]. However, the authors were able to show that about 35% of patients experienced a change in living disposition postoperatively. Thus, the authors conclude that understanding the rates and risk factors for adverse events following glioblastoma resection should guide neurosurgeons in treatment decision in this selected patient cohort [27]. Here, we propose the mFI is quite an easy to use screening method that not only enables to preoperatively select for high-risk patients that might require special attention in surgical management and after care, but also might become crucial for optimizing individually tailored treatment strategies as well as counselling of patients and family members. Further multicenter-based studies are needed in order to become able to sufficiently cope with the challenges in the course of interdisciplinary modern treatment and aftercare in the geriatric glioblastoma patient cohort.

## Limitations

The present study has multiple limitations. The data collection was conducted retrospectively. The patients were not randomized, but treated according to the preference of the treating physicians. Furthermore, the study utilizes the mFI as an assessment tool for frailty, which does not query all established factors of frailty. Nevertheless, the strength of the present work is found in the detailed, standardized collection of these data, the results of which should be supported by the initiation of multicenter prospective studies.

## Conclusions

The present study is the first to demonstrate a distinct association between preoperatively increased frailty and decreased survival following surgical treatment of geriatric glioblastoma patients. The results of this study should contribute to a better assessment of the risk/benefit ratio for the treating physician, so that a different kind of focused attention during treatment course might arise and both patients and relatives might be better informed prior surgery. Thus, the authors emphasize that comprehensive assessment of geriatric patients with glioblastoma, including comorbidity burden, frailty and nutritional status, may help clinicians to develop suitable, appropriate and precise treatment strategies for these vulnerable patients.

## Electronic supplementary material

Below is the link to the electronic supplementary material.

Supplementary file1 (tif 4635 kb)
