# Supplementary figures and images for: Newly diagnosed glioblastoma in geriatric (65 +) patients: impact of patients frailty, comorbidity burden and obesity on overall survival
Source: J Neurooncol. 2020 Sep 29;149(3):421–7. doi: 10.1007/s11060-020-03625-2 (PMC7609438; doi:10.1007/s11060-020-03625-2)

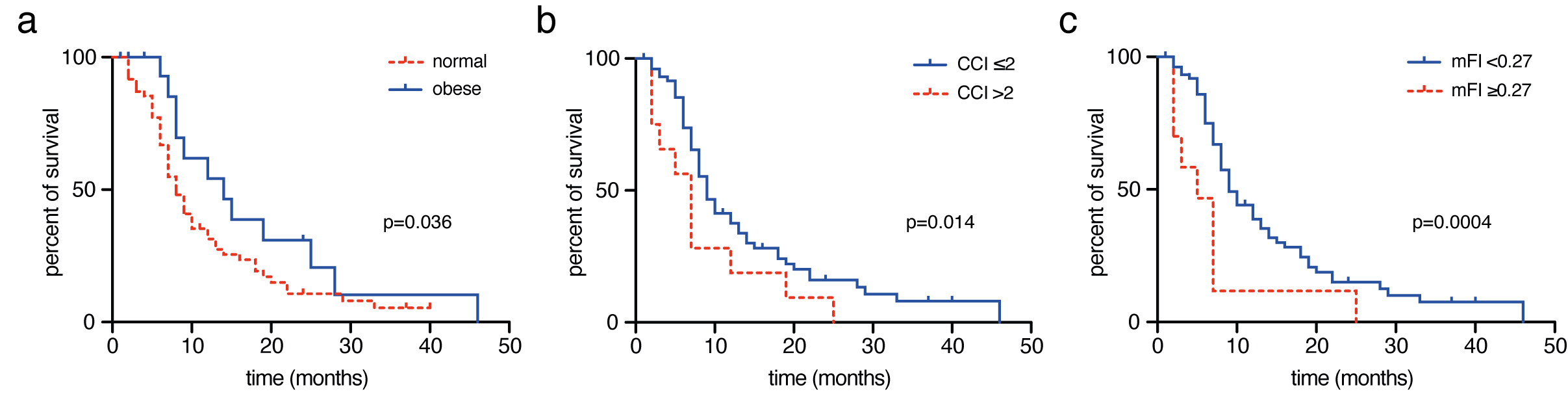

Supplement: Supplementary file 1 — Supplementary file1 (tif 4635 kb) [file 11060_2020_3625_MOESM1_ESM.tif]
